# Dopaminergic modulation of positive expectations for goal-directed action: evidence from Parkinson’s disease

**DOI:** 10.3389/fpsyg.2015.01514

**Published:** 2015-10-08

**Authors:** Noham Wolpe, Cristina Nombela, James B. Rowe

**Affiliations:** ^1^Department of Clinical Neurosciences, University of CambridgeCambridge, UK; ^2^Medical Research Council Cognition and Brain Sciences UnitCambridge, UK; ^3^Behavioural and Clinical Neuroscience Institute, University of CambridgeCambridge, UK

**Keywords:** positive expectation, voluntary action, agency, Parkinson’s disease, dopamine, Bayesian, placebo, inverted U-shaped function

## Abstract

Parkinson’s disease (PD) impairs the control of movement and cognition, including the planning of action and its consequences. This provides the opportunity to study the dopaminergic influences on the perception and awareness of action. Here we examined the perception of the outcome of a goal-directed action made by medicated patients with PD. A visuomotor task probed the integration of sensorimotor signals with the positive expectations of outcomes (Self priors), which in healthy adults bias perception toward success in proportion to trait optimism. We tested the hypotheses that (i) the priors on the perception of the consequences of one’s own actions differ between patients and age- and sex-matched controls, and (ii) that these priors are modulated by the levodopa dose equivalent (LDEs) in patients. There was no overall difference between patients and controls in the perceptual priors used. However, the precision of patient priors was inversely related to their LDE. Patients with high LDE showed more accurate priors, representing predictions that were closer to the true distribution of performance. Such accuracy has previously been demonstrated when observing the actions of others, suggesting abnormal awareness of action in these patients. These results confirm a link between dopamine and the positive expectation of the outcome of one’s own actions, and may have implications for the management of PD.

## Introduction

Parkinson’s disease (PD) is a common neurodegenerative disease, associated with the loss of dopaminergic projections from the substantia nigra and ventral tegmentum to the striatum and frontal cortex respectively (reviewed in Cools, 2006). PD causes a disorder of movement with tremor, rigidity, and bradykinesia (Hughes et al., 1992). However, it also affects motor cognition, including executive function such as planning, sequencing and initiating movements (Owen et al., 1992; Williams-Gray et al., 2007a; Hughes et al., 2010, 2013). Dopamine replacement therapies alleviate some of these cognitive functions and their underlying neural circuits, but impair others (Gotham et al., 1988; Kehagia et al., 2010; Rowe et al., 2010).

Parkinson’s disease can also change the perception and awareness of voluntary action. For example, the perception of the position and motion of one’s own body parts, known as ‘kinaesthesia,’ is impaired by PD (Klockgether et al., 1995), possibly due to an abnormal processing of sensory feedback (Konczak et al., 2012). Levodopa alleviates kinaesthetic deficits in some studies (Li et al., 2010), but not in others (O’Suilleabhain et al., 2001).

The effect of PD on the perception of self-generated actions was also assessed by the intentional binding paradigm. Intentional binding refers to the perceived temporal attraction between a voluntary action and its sensory effect in instrumental behavior (Haggard et al., 2002). It has been used as an objective measure for the awareness of action and sense of agency (Wolpe and Rowe, 2014). This temporal attraction is unchanged in PD patients, but is enhanced by levodopa (Moore et al., 2010).

Together, these inconsistent results demonstrate a complex effect of PD and levodopa on the awareness of action, which might reflect a change in the expectations of outcomes rather than changes in sensation *per se*. We therefore studied the effect of PD and levodopa on the positive expectations of the outcome of goal-directed actions.

We recently reported that people’s perception of the outcome of their own goal-directed action is normally biased toward goal success (Wolpe et al., 2014). The bias can be explained by optimistic Bayesian Self priors – that is, the exaggerated reliability of expectations of success set by the goal of the action, and integrated with the sensorimotor signals for perception. The priors are optimistic in the sense that they have a narrower distribution around the intended goal, relative to the actual distribution of performance. In contrast, for observed actions people use priors that more accurately represents the observed performance (Wolpe et al., 2014).

Optimistic expectations are normally integrated with sensorimotor signals that contribute to the awareness of action (Frith et al., 2000). For example, a cognitive process can ‘exaggerate’ the reliability of low-level sensorimotor prediction signals, such as the efference copy of the motor command (Wolpert and Ghahramani, 2000). The integration leads to narrow Self priors that support the correct attribution of actions by facilitating the difference between perception of one’s own and others’ actions (Wolpe et al., 2014).

Dopamine is a key neuromodulator that signals reward expectation in the striatum during operant action-reward conditioning (de la Fuente-Fernández et al., 2001). Striatal dopamine loss might therefore alter the positive expectation of outcomes of one’s actions. Moreover, the representation of expected reward in medial frontal cortex is also dependent on dopamine (Rowe et al., 2008), and liable to hyper-dopaminergic effects in early PD (Rakshi et al., 1999) as well as dopamine loss in later stages.

The current study sought to examine the effect of dopamine on the perception of the outcome of goal-directed action. We tested the specific hypotheses that PD and levodopa modulate the perception of action by changing the exaggeration of expected performance (optimism). Patients in mild to moderate stages of PD participated, on their usual medication. We hypothesized that (i) PD would diminish the positive expectations for goal-directed actions, represented in reduced reliability of Self priors; and that (ii) the levodopa dose equivalents (LDE) of dopaminergic medication would determine the reliability of these priors and alter the perception of one’s goal-directed actions.

## Materials and Methods

### Participants

Twenty patients (13 men; aged 48–81 years mean: 68; SD: 10) were recruited from the John van Geest Centre for Brain Repair, PD research clinic. Patients met clinical diagnostic criteria of idiopathic PD, according to the UK PD brain bank criteria (Hughes et al., 1992), and were mild to moderate stages of disease, [Hoehn and Yahr stages 1–3] (Hoehn and Yahr, 1967). In addition, 20 age- and sex-matched, neurologically healthy controls, (12 men; aged 55–76 years, mean: 68; SD: 6) were included in the study, and were compensated with £12 for their participation. All subjects were right-handed, and gave written informed consent before the experiment. The study was approved by the Cambridge Research Ethics Committee.

Assessment of motor and cognitive symptoms in patients was performed at the beginning of the testing session. The severity of motor features was assessed with the Unified Parkinson’s Disease Rating Scale (UPDRS) motor subscale III (Fahn and Elton, 1987). Cognitive abilities in both patients and controls were assessed through the Mini-Mental State Examination (MMSE; Folstein et al., 1975) and Addenbrooke’s Cognitive Examination Revised (ACE-R; Mioshi et al., 2006). Cognitive impairment was an exclusion criterion: as ACE-R has better sensitivity and specificity for cognitive impairment in PD than MMSE (Reyes et al., 2009), we used the ACE-R cut-off score of 83/100 (Reyes et al., 2009).

All patients were on dopamine replacement therapy at the time of the experiment: patients were tested in the morning after taking their morning medication as normal. The time interval between levodopa self administration and testing varied between 1 and 3 h, such that patients were in a relative ‘on’ state. Our patients had mild to moderate PD (see **Table 1**) and were not affected by clinically significant on-off phenomena or freezing. LDE was computed according to Tomlinson et al. (2010).

**Table 1:** Demographic details of Parkinson’s disease (PD) patients participating in the study.

No.	Gender (M/F)	Age	Disease duration (years)	Disease stage^∗^ (0-5)	UPDRS-III motor subscale (0-56)	MMSE (0-30)	ACE-R (0-100)	LDE ^∗∗^
1	M	74	16	2	20	30	98	620
2	F	76	10	2	26	29	91	560
3	M	81	15	2.5	13	29	94	410
4	M	54	15	1	13	29	89	1817
4	F	73	13	2.5	16	28	95	565
6	F	76	9	3	37	30	91	855
7	F	81	13	2	15	29	97	1355
8	M	77	13	2	24	30	97	1722
9	M	64	6	1.5	21	29	94	1175
10	M	48	6	2.5	16	29	88	536
11	M	72	26	1	21	28	85	460
12	M	69	10	1	21	23	84	276
13	M	57	14	1	31	28	94	1180
14	F	66	17	2	19	27	95	1740
15	M	76	12	1	18	27	87	1500
16	F	64	11	3	16	28	96	1000
17	M	77	9	3	19	27	88	700
18	F	55	8	2	20	29	98	200
19	M	56	11	3	14	29	86	1210
20	M	63	11	1.5	16	29	94	1315
** Mean**		68	12	2	20	28	92	960

*UPDRS, Unified Parkinson’s Disease Rating Scale; MMSE, Mini-Mental State Examination; ACE-R, Addenbrooke’s Cognitive Examination Revised; LDE, Levodopa Dose Equivalent; ^∗^According to Hoehn and Yahr (1967); ^∗∗^Calculated according to Tomlinson et al. (2010).*

### Self Prior Task and Procedure

**FIGURE 1:**
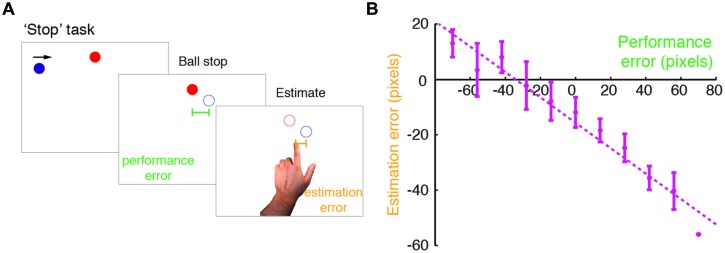
**Modified ‘Stop’ task performed in the study. (A)** An illustration of the modified Self Stop task (Wolpe et al., 2014). Subjects watched a blue ball that repeatedly swept horizontally across the screen in a rightward motion. They were asked to press a key with their right hand, so as to stop the ball when it was vertically aligned with a red target. Following the stopping event, subjects estimated where they had stopped the ball, by pointing on the screen with their left index finger. For each trial, we examined their performance error (difference between the actual stopping position and the target position) and estimation error (difference between the pointing position and the actual stopping position). **(B)** Estimation errors plotted against performance errors for a typical subject (here a control subject). The dashed line indicates a linear regression fit, and the error bars indicate SD. Note the leftward shift in the intercept of the regression line, which was accounted for in the Bayesian model fitting.

Subjects performed a modified version of the Self Stop task described in Wolpe et al. (2014) (**Figure 1A**). Subjects were seated 0.5 m from a 17′ Protouch LCD touch screen with 1024 × 768 resolution (26 pixels/cm) that refreshed at 60 Hz. All stimuli were displayed with Matlab Psychophysics toolbox (Brainard, 1997). In brief, subjects were asked to stop a blue ball (15 pixel radius), which repeatedly swept the screen horizontally in a rightward motion. When it was vertically aligned with a red circle target (15 pixel radius), subjects pressed a key with their right index finger to stop the ball. The ball vanished following the key press, and 250 ms later the target disappeared. The ball’s starting position was randomized across trials (drawn from a uniform distribution covering the horizontal extent of the screen). The target was horizontally centered, and was displayed close to the top of the screen, just above the sweeping blue ball. The number of ball sweeps was limited to three, in order to control for stopping times across groups, thereby minimizing potential group differences in task demands. When failing to respond after three sweeps, the trial was stopped, and before it was restarted subjects received a message on the screen prompting them to respond faster.

Subjects were asked to point with their left index finger on the screen where the ball had stopped (i.e., final position before vanishing). Screen pointing was used after a pilot experiment showed that it was preferable to using a mouse for many patients. The left hand was chosen to facilitate fast responses, as the right hand remained resting on the key in preparation for the next trial. The spatial shift in pointing due to hand use was corrected in the analysis by including an additional spatial shift parameter (see *Analyses*).

Subjects were also given the option to skip the trial during the estimation of the stopping point, by pointing at the word ‘Skip’ displayed at the bottom of the screen. Any trial that was skipped was excluded, and an additional trial was added instead, so that all subjects’ dataset had an equal number of trials (on average four trials skipped per subject). The experiment was performed in blocks of trials. The experiment started with a short practice block of 12 trials to familiarize subjects with the task. Following practice, two blocks of 52 trials each were performed.

### Analyses

As in Wolpe et al. (2014), for each trial we calculated the estimation error (distance between estimated stopping position and true stopping position) and the performance error (distance between true stopping position and target). For each subject, we first fitted a linear regression of estimation errors against performance errors to examine any bias in estimation. In order to have a consistency in the estimation procedure and to minimize a possible interference from different memory processes, we excluded outlier trials with longer estimation times in the following manner: trials with estimation times greater than 2 SDs from the mean were excluded (on average one trial for each control and four trials for each patient). One additional patient was excluded from the study cohort, as his mean estimation time was larger than 3 SDs from the patient group mean.

We inferred the subjects’ priors by fitting Bayesian models through maximum likelihood estimation – that is, maximizing the probability of each subject’s dataset. We used a constrained model, in which the mean of priors was centered on the target, and the mean of sensory evidence was centered on the true stopping position. However, as subjects used their left hand to estimate the stopping position, we expected there would be a consistent spatial (left) shift in estimates that should be accounted for in the model.

Such a spatial shift might be incorporated in the prior mean, in the visual evidence mean or in both. In the non-linear model fitting of the maximum likelihood estimation process, a model with a shift in the prior mean; a model with a shift in sensory evidence mean; and a model with a shift in both prior and sensory evidence, are all mathematically equivalent and will converge on the same parameters. However, a model that includes a shift in both prior and sensory evidence has an additional free parameter compared to only a shift in either. Moreover, we expected the spatial shift to arise due to the screen pointing process and independent of the prior. We therefore used a model with a shift in evidence mean (which in this experiment includes both sensory noise and noise added during the screen pointing) in addition to the constrained model above. We used the following equation derived in Wolpe et al. (2014):

$$x_{\mathrm{estimate}}=w*{\bar{x}}_{\mathrm{prior}}+(1-w)*({\bar{x}}_{\mathrm{evidence}}+{\mathrm{shift}}_{\mathrm{evidence}})$$

with 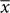
_prior_ centered on the target, and the weighting w given by:

$$w=\frac{\sigma_{\mathrm{evidence}}^{2}}{\sigma_{\mathrm{prior}}^{2}+\sigma_{\mathrm{evidence}}^{2}}(Ghahramani et al., 1997).$$

To summarize, we fitted the following models:

1) Constrained model, with only prior SD and visual evidence SD as free parameters (2 parameters).
2) Prior SD and visual evidence SD as well as a spatial shift in visual evidence (3 parameters).

The model that best explained the data was selected using the Bayesian Information Criterion (BIC) with a threshold difference of 6 for ‘strong’ evidence (Schwarz, 1978; Raftery, 1995). Only parameters of the winning models were presented. For correlations with the clinical data and age, non-parametric Spearman’s correlations were performed.

## Results

The clinical details of patients are summarized in **Table 1**. Patients had a mixed severity of motor features and duration of illness, but they were all in mild to moderate stages of disease (Hoehn and Yahr, 1967).

Patients and controls were all able to perform the modified ‘Stop’ task (**Figure 1A**). Our first objective was to examine the difference in the perception of the consequences of one’s own goal-directed actions between PD patients and controls. To this end, we compared the perceptual bias toward the target measured in the regression slopes, followed by a comparison of the Bayesian model parameters.

Before examining the perceptual bias and priors, we first compared the time it took subjects to complete the task as a possible confound. Patients and controls did not differ in the time required for stopping the ball (*t*_38_ = -0.69, *p* = 0.49). In contrast, there was a group difference in the time to estimate the ball stopping position (*t*_38_ = 2.09, *p* = 0.043). Mean Estimation time in patients was 1.07 s, but only 0.88 s in controls. We therefore included mean estimation times as a nuisance covariate in both the between-group and within-group analyses of the Bayesian priors below.

### Perceptual Biases Toward the Target Across Groups

We examined subjects’ bias toward the target, by fitting a linear regression of estimation errors against performance errors for each subject (**Figure 1B**). Mean intercept (i.e., ball stopping position) in patients was just six pixels left of the target, and 15 pixels left of the target in controls (see below). Estimation errors were always biased in a graded manner, as measured by a negative regression slope, for both patients (slope smaller than zero in patients: *t*_19_ = -9.83, *p* < 0.001) and controls (*t*_19_ = -8.16, *p* < 0.001). The extent of the bias, in terms of the regression slope, was not different between patients and controls (*t*_38_ = -1.277, *p* = 0.419; Bonferroni corrected).

### Comparison of Bayesian Model Parameters Across Groups

We next fitted the data with two Bayesian models: (1) a model with SDs of sensory evidence and prior, constrained with the prior centered on the target and the evidence centered on the true stopping position; (2) a model with SDs of evidence and prior, and a spatial shift on evidence, likely to arise due to the estimation procedure of using the left hand (see **Figure 1B**). On a group-level, average difference in BIC was only 1.4 in favor of model 2. However, on an individual-subject level, model 2 was more likely than model 1 in 29/40 subjects (BIC difference greater than 6 in 12/20 patients and 17/20 controls), which was significant by a sign test (two-tailed; *p* = 0.006). We therefore report the parameters of model 2.

Examining the model parameter of shift from stopping position in the distribution of sensory evidence, subjects had a consistent leftward shift on their sensory evidence (*t*_38_ = -4.8, *p* < 0.001). This shift is likely to have resulted from using the left hand during the screen pointing procedure. Importantly, the shift did not differ across groups (mean shift in patients: 18 pixels; mean shift in controls: 23 pixels; *t*_38_ = 0.95, *p* = 0.35; uncorrected).

Examining the distribution of sensory evidence, there was a significant increase in the SDs of sensory evidence in patients compared to controls (*t*_38_ = 3.14, *p* = 0.003). This increase in patients could reflect an elevated sensory noise, or more noise attributed to the estimation procedure. Our main focus in this study, however, was on the differences in priors between patients and controls and within the patient group with relation to levodopa.

**FIGURE 2:**
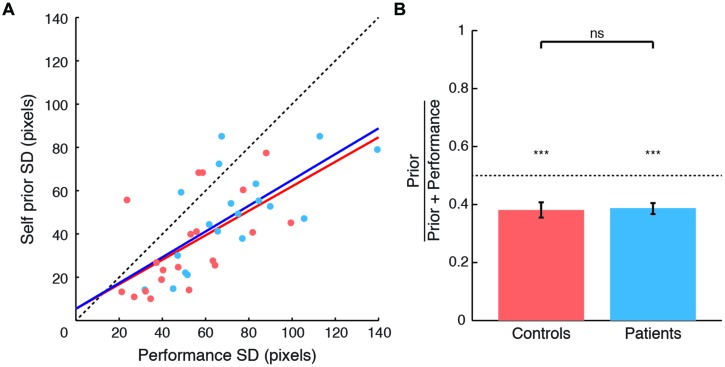
**Comparison between Self priors across groups. (A)** SDs of Self priors plotted against SDs of performance errors for patients (blue circles and regression line) and controls (orange circles and regression line). Data points lie below the line of equality (black dashed line) in 34 of the 40 subjects. **(B)** Prior SDs normalized to performance error SDs, plotted for patients (blue) and controls (orange). For both groups, the SDs of priors were smaller than the SDs of performance errors. No difference was found across groups. Significance level indicated by ^∗∗∗^*p* < 0.001.

The SDs of priors correlated with performance error SDs for both patients (*r* = 0.706, *p* < 0.001) and controls (*r* = 0.582, *p* = 0.007) (**Figure 2A**), as in Wolpe et al. (2014). Based on this strong correlation, and as patient performance error SD was greater than that in controls (*t*_38_ = 2.33, *p* = 0.025), we examined priors with their values normalized to performance distribution (**Figure 2B**). These normalized priors reflect the degree of exaggeration of positive expectations for each subject. SDs of priors for both patients and controls were significantly smaller than SDs of performance distribution (normalized values smaller than 0.5 for patients: *t*_19_ = -5.92, *p* < 0.001; and controls: *t*_19_ = -4.482, *p* < 0.001). These results replicate the exaggerated Self priors found in Wolpe et al. (2014).

As patients and controls showed a significant difference in estimation time (see above), we included estimation time as a nuisance covariate in the comparison across groups in an analysis of covariance. Normalized priors did not differ across groups (**Figure 2B**), with very small effect sizes: there was neither a main effect of group [*F*_(1,36)_ = 0.063, *p* = 0.803, η^2^ = 0.002], or estimation time [*F*_(1,36)_ = 0.081, *p* = 0.777, η^2^ = 0.002], nor a group × estimation time interaction [*F*_(1,36)_ = 0.12, *p* = 0.731, η^2^ = 0.003]. An additional exploratory analysis showed no correlation between prior width and disease severity as measured by UPDRS motor subscale (Spearman’s ρ = -0.01, *ns*). Together, these results suggest that PD patients demonstrated a normal group-average prior despite the likelihood of individual differences in disease and treatment. We next explored the source of within-patient variability, by examining the relation between priors and LDE.

### Relation between Priors and Levodopa

**FIGURE 3:**
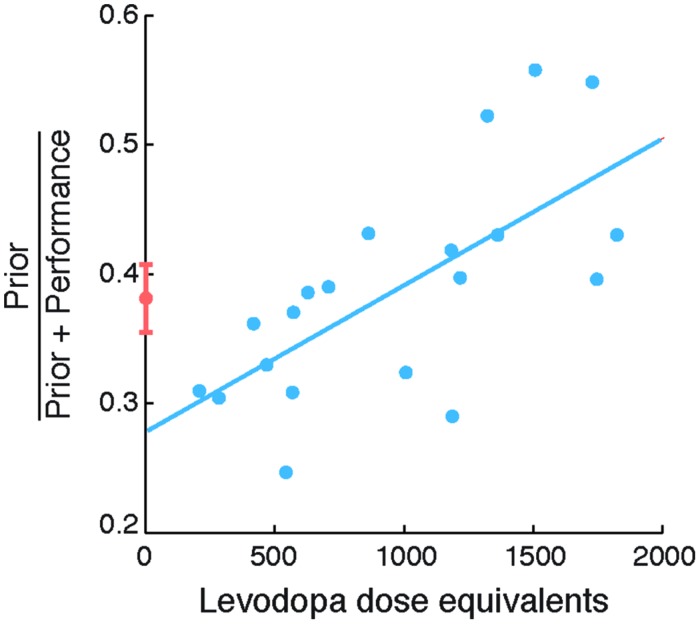
**Self priors versus levodopa dose equivalent in patients with Parkinson’s disease (PD).** Normalized Self priors plotted against levodopa dose equivalents in patients (blue circles). The blue line indicates a linear regression for illustration purposes. Controls’ data are plotted for reference (orange circle and error bars, indicating control group mean and standard error of the mean, respectively).

The second objective of our study was to examine the relationship between the degree of exaggeration in priors and its relationship to levodopa doses in PD patients. Specifically, we examined the relation between individual differences in Self priors and LDEs (**Figure 3**). We found a strong correlation between normalized prior SDs and LDEs (Spearman’s ρ = 0.723, *p* = 0.002), arising from a significant correlation between prior SDs and LDEs across patients (Spearman’s ρ = 0.585, *p* = 0.039). The correlation was positive, indicating that patients with higher LDE had wider priors.

The correlation between normalized priors and LDEs remained significant even after accounting for disease duration (partial correlation; Spearman’s ρ = 0.723, *p* = 0.002); severity of motor features measured using the UPDRS motor subscale III (partial correlation; Spearman’s ρ = 0.76, *p* < 0.001); or subject mean estimation time (partial correlation; Spearman’s ρ = 0.746, *p* = 0.001; with Bonferroni correction). For completeness, we note that there was no consistent relation between the SDs of sensory evidence and LDEs (Spearman’s ρ = 0.32, *p* = 0.17). Taken together, these results suggest that the extent of exaggeration of the reliability of Self priors in patients was strongly related to levodopa doses.

The correlation between the extent of exaggeration of Self priors and levodopa in patients suggests that patients on higher levodopa had priors that were closer to the true performance distribution. However, the ‘accuracy’ of priors is not linearly related to the values of normalized priors, which can vary between 0 and 1, but would be 0.5 in the case when priors and performance error SDs are equal. To formally test how the accuracy of priors is related to levodopa, we therefore correlated the absolute difference between normalized priors and 0.5 with levodopa doses. A strongly significant negative correlation emerged (Spearman’s ρ = -0.717, *p* = 0.001; Bonferroni corrected). This negative relation suggests that patients with high levodopa doses used priors that deviated less from the distribution of performance, and were thus more accurate, similar to the perception of observed actions (Wolpe et al., 2014).

## Discussion

The principal results of this study are that (i) the group-mean of the positive distortion of perceived outcomes of one’s own goal-directed action was similar in medicated PD patients and healthy individuals; but (ii) there is a strong correlation between dopaminergic dose and the degree of positive exaggeration of priors in patients. This correlation indicates that patients on higher levodopa doses more accurately perceive the outcome of their own actions, in a way that healthy people perceive the actions of others but not of themselves.

### Group Comparison of Self Priors

Although Self priors were related to dopaminergic medication, there was no overall group difference. We suggest that this reflects the dynamic effects of disease on basal ganglia function. For example, in early stages of PD, the dorsal regions of the basal ganglia that are implicated in habitual actions are most affected, while ventral regions that are most associated with goal-directed control are largely preserved (Redgrave et al., 2010). As a result, goal-directed behaviors gradually replace or compensate for the impairment of automated movements based on stimulus-response associations (Redgrave et al., 2010; Torres et al., 2011). As patients included in our study had mild to moderate disease (median Hoehn and Yahr stage two), we suggest that goal-directed actions and their perception remained relatively intact.

Another feature of abnormal motor control in PD is the increased reliance of patients on external cues. The significance of this feature is underscored in the common clinical observation of improved gait and postural stability in PD patients following sensory cueing (Azulay et al., 2002, 2006). Several studies have demonstrated the strong influence of visual feedback on patient movements (Flash et al., 1992; Klockgether and Dichgans, 1994; Schettino et al., 2006), which may partially compensate for kinaesthetic impairments (Azulay et al., 2006). When visual feedback is occluded, patients tend to be slower and less accurate than when it is available (Klockgether and Dichgans, 1994; Schettino et al., 2006).

Our visuomotor task could reinforce the tendency of patients to over-rely on visual cues. As it disappears just before the estimation phase of the task, the target potentially provides patients with a visual cue to help them complete the estimation task more quickly and accurately. Bias toward the target as a visual cue for the estimation procedure may offset the diminution of priors. This behavioral feature of PD is unlikely to account for the relationship between levodopa and the width of priors, as levodopa does not significantly affect the reliance on external cues for movement (Burleigh-Jacobs et al., 1997). In addition to the altered integration of visual signals in PD, impaired oculomotor function (e.g., Shibasaki et al., 1979; Perneczky et al., 2011) could influence performance on our task. However, the absence of a group difference in our task and the minimal effect of levodopa on oculomotor function (Corin et al., 1972) suggest that the effect of oculomotor dysfunction on our principal results is minimal.

Although we have focussed on the degree of exaggeration of sensorimotor prediction for the perception of action, it is worth noting that previous studies have suggested preservation of other sensorimotor prediction signals in PD. For example, intentional binding, the perceived temporal attraction between a voluntary action and its sensory effect, is dependent on an intact sensorimotor prediction (Waszak et al., 2012; Wolpe et al., 2013; Wolpe and Rowe, 2014), but is unaffected in PD (Moore et al., 2010). Moreover, kinaesthetic deficits in PD have been found to be driven by abnormal low-level sensory processing, rather than changes in prediction processes (Konczak et al., 2012). Similarly, we found increased noise on sensory evidence in patients, which can be attributed to abnormal processing of sensory input or to a greater noise in the motor output during the estimation process.

A second potential contributor to the increased sensory noise shown by patients is the time it took patients to complete the procedure. On average, patients were 200 ms slower than controls in estimating the sensory consequences of their actions, although they were not slower in their response time for stopping the ball. This result is perhaps not surprising, considering the bradykinesia that is characteristic of PD, but simple motor response times are not necessarily increased in PD and the additional estimation time may indicate an impairment in the cognitive decision processes. The estimation of time or temporal intervals is also affected by PD (Pastor et al., 1992; Buhusi and Meck, 2005). This might have been expected to influence the perception of velocity of the moving ball, or the horizontal distance traveled during, although neither the simple regression models nor Bayesian models to estimate the Self priors suggested a group-wise difference even when accounting for the different estimation times. However, PD is a heterogeneous disorder and in the next section we consider the causes of individual differences in priors.

### A Relation between Self Priors and Levodopa Dose

The marked individual differences in our patients (see **Figure 3**) is typical of studies of heterogeneous neurodegenerative disorders like PD. This variation is in part due to individual differences in the extent and severity of striatal dopaminergic denervation and cortical involvement (e.g., Cools, 2006; Rowe et al., 2008). Much of the variability in our patients’ priors was explained by LDE (Spearman’s ρ 0.723), which positively correlated with the degree of exaggeration in priors. Patients with higher levodopa doses demonstrated more accurate Self priors that are more similar to the priors used for observing others’ actions (Wolpe et al., 2014).

We suggest that exogenous dopamine in PD (indexed by the LDE) is related to positive expectations: patients under higher levodopa doses do not show the normal exaggeration of Self priors. However, the effect of dopamine on fronto-striatal functions in PD is often described as an inverted U-shaped curve, such that level of baseline dopaminergic function determines whether dopaminergic medication enhances or impairs cognitive functions (Rowe et al., 2008; Wu et al., 2012; Hughes et al., 2013). These baseline levels are in part determined by the differences in the integrity of dopaminergic innervation in the parallel corticostriatal circuits for motor, oculomotor, limbic and cognitive function. There are also individual differences due to polymorphism in the genotype of Catechol *O*-Methyltransferase enzyme (Williams-Gray et al., 2007b; Nombela et al., 2014). In our study, the correlation between the width of Self priors and LDE was centered on the mean of controls (no group difference), confirming that low levodopa doses have the opposite effect to high doses in PD patients, a result of inverted U-shape dose-response relationships (Cools and D’Esposito, 2011).

This U-shaped relation suggests that low doses of levodopa might preserve, or even further enhance the precision of priors, leading to increased optimistic expectations. It has been shown that a single administration of levodopa in young healthy adults can enhance hedonic expectations for future events (Sharot et al., 2009). Subjects gave higher ratings of prospective ‘happiness’ to different vacation destinations when given levodopa compared to placebo. The levodopa dose used by Sharot et al. (2009) was 100 mg, which according to our data might lead to such narrower priors (see Figure 5.3A) and the related enhancement in optimism bias. Taken together, these results confirm the dose-dependent effect of dopaminergic medication on goal priors and the resulting optimism bias. It also means that levodopa administration in young healthy adults cannot be equated to older neurodegenerative patients in which the dopaminergic systems are severely perturbed at baseline.

As the narrow Self priors might support the normal attribution of action (Wolpe et al., 2014), these results imply that PD patients on high levodopa doses have an impaired sense of agency. Interestingly, levodopa treatment in PD has indirectly been shown to alter the sense of agency, increasing overall intentional binding of action and its effect (Moore et al., 2010). Together, these findings not only suggest an impaired awareness of action in medicated PD patients, but also support the application of Self priors as an objective measure of agency processes (Wolpe and Rowe, 2014).

### The Mechanism of Action of Dopamine on Self Priors

Both cognitive and sensorimotor processes are required for generating predictions for the perception of one’s goal-directed action (Wolpe et al., 2014). For example, there is a prediction of the ensuing sensory effect from the efference copy of the motor command using a forward model; a process which is optimized by learning the relation between an action and its sensory effect (Wolpert and Ghahramani, 2000). These predictions might be adjusted according to prior knowledge about the world, for example the distribution of one’s motor performance (Körding and Wolpert, 2004). The reliability of this low-level sensorimotor prediction can be ‘exaggerated’ by top–down cognitive mechanisms, such as conscious expectations (Sterzer et al., 2008), motivational states (Balcetis and Dunning, 2006) and the illusions of superiority (Wolpe et al., 2014).

The role of striatal dopamine in such prediction processes has been established in health (Pessiglione et al., 2006). In medicated PD patients, the relation between the degree of exaggeration measured in Self priors and dopamine could stem from either the aberrant endogenous dopamine release, or the influx of exogenous dopamine from the levodopa treatment. We next discuss these alternative mechanisms.

Dopamine release in the nigrostriatal system in PD has been linked to the strength of placebo effect – that is, the clinical improvement following a non-active treatment due to the positive expectation of benefit (de la Fuente-Fernández et al., 2001). Increased nigrostriatal damage may lead to diminished positive expectations, thereby reducing the placebo effect in PD (de la Fuente-Fernández et al., 2001). This link can also explain the relation between Self priors and dopamine in our study: as levodopa doses are tailored for each patient’s motor impairments, the computed LDEs may, at least in part, be regarded as a proxy for nigrostriatal integrity. Patients on higher levodopa doses, suggesting greater nigrostriatal damage, would thus show impairment in the normal positive expectations reflected in the narrow Self priors, resulting in a more ‘accurate’ and less optimistic perception.

However, levodopa therapies can have detrimental effects on patient cognition (Cools, 2006), due to the uneven distribution of striatal and cortical dopaminergic pathology in PD. Dorsal striatum is most affected early, while ventral striatum and mesocortex are relatively intact in early disease stages (reviewed in Kish et al., 1988; Kehagia et al., 2010). As patients’ levodopa doses are usually adjusted according to the motor deficits (closely related to the dorsal striatum), they can cause a dopaminergic overdose of the ventral striatum system (Cools, 2006). Areas connected to the ventral striatum and the mescortical system, including anterior cingulate and ventromedial cortex, are hyperdopaminergic in early medicated PD (Rakshi et al., 1999; Wu et al., 2012). The anterior cingulate and ventromedial cortex have been implicated in the illusions of superiority (Beer and Hughes, 2010) and optimism bias (Sharot et al., 2007), which can be explained in terms of exaggerated Self priors (Wolpe et al., 2014). In our study, increasing doses of levodopa could have thus impaired the exaggeration of reliability of sensorimotor prediction by overdosing the mesocortical pathway.

### Implications for PD

In healthy adults, the degree of exaggeration in Self priors has been shown to be associated with trait optimism bias: people who show wider priors tend to be less optimistic (Wolpe et al., 2014). The exaggerated priors and the related ‘positive’ cognitive illusions could thereby facilitate motivation and adaptive behavior (Taylor and Brown, 1988). In PD patients, however, increasing damage to nigrostriatal circuits and/or long-term dopaminergic overdose of the mesocortex impair the exaggeration of the reliability of priors, leading to a more accurate but pessimistic perception of one’s actions. The persistence of this pessimistic and accurate perception might lead to a reduced motivation as a result of a ‘depressive realism’ (Alloy and Abramson, 1988). The abnormally accurate perception of the consequences of ones actions does not imply accurate choices in the selection of actions. Indeed, dopaminergic dysregulation in PD alters value based decision making (Voon et al., 2010), which predisposes to impulsive and addictive behaviors (Napier et al., 2014).

A more accurate perception of the outcomes of one’s actions might put patients at risk for depression and poor motivation, which are indeed often observed in PD (e.g., Gotham et al., 1986); and which contribute to the reduced quality of life in patients (Schrag et al., 2000). Similarly, broadening of Self priors might reflect diminished positive expectations, including reduced expectation of benefit from treatment (de la Fuente-Fernández et al., 2001), impairing the alleviation of symptoms.

The administration of dopamine in PD can *elevate* mood and reduce anxiety (Maricle et al., 1995; Czernecki et al., 2002). Critically, the reported improvement in depressive symptoms in such studies was the result of a short-term levodopa administration following overnight withdrawal and were measured using self-reports and questionnaires (Maricle et al., 1995; Czernecki et al., 2002). However, the effect of dopamine on patients withdrawn from chronic dopaminergic treatment could be due to the alleviation of acute withdrawal effects, rather than indicating a long-term role in behavior (Nestler, 2001). In contrast, our study measured sensorimotor and perceptual processes while the patients were on their usual medication.

The current study has several limitations. Firstly, we do not separate our analysis according to the laterality of dominant motor symptoms. This varied across patients, and could affect the motor performance on the visuomotor task, and may add unexplained variability to the correlation analyses. However, in the absence of *a priori* hypotheses of lateralised processes, small numbers and the presence of a systemic therapy, we suggest that collapsing across the factor of laterality is preferable. In addition to altered control of the hands which may affect performance in the task, their impaired oculomotor function (Corin et al., 1972; Shibasaki et al., 1979) may have also compromised patient performance of the visuomotor task.

In light of the demands of the visuomotor task and to facilitate compliance, patients were only tested whilst on their usual dopaminergic medication. We did not measure their perception of action ‘off’ medication. Moreover, within each patient we did not test across drug phases of their on–off cycle, i.e., in different times relative to levodopa administration. We could not therefore dissociate the possible mechanisms through which dopamine modulates Self priors in PD; and could not establish a causal effect of dopamine on Self priors as our results are correlational.

## Conclusion

For the perception of one’s own goal-directed actions, the use of exaggerated priors persists in PD. However, the dopaminergic dose was negatively correlated with the degree of exaggeration of priors, such that patients on more dopaminergic medication had more accurate priors that closely represented the true distribution of performance. This veridical perception is normally only present for the observation of others’ actions. Our results suggest that positive expectations can be increasingly impaired in PD, especially with a relative dopaminergic overdose of the ventral striatum and mesocortical systems. The resulting changes in perception of action outcomes have implications for understanding the normative mechanisms, and the risks of affective and behavioral symptoms in PD.

## Conflict of Interest Statement

The authors declare that the research was conducted in the absence of any commercial or financial relationships that could be construed as a potential conflict of interest.
